# Large Outbreak of Coronavirus Disease among Wedding Attendees, Jordan

**DOI:** 10.3201/eid2609.201469

**Published:** 2020-09

**Authors:** Dawood Yusef, Wail Hayajneh, Samah Awad, Suleiman Momany, Basheer Khassawneh, Shaher Samrah, Basil Obeidat, Liqaa Raffee, Ibrahim Al-Faouri, Ali Bani Issa, Heba Al Zamel, Enas Bataineh, Reem Qdaisat

**Affiliations:** Jordan University of Science and Technology, King Abdullah University Hospital, Irbid, Jordan

**Keywords:** respiratory infections, severe acute respiratory syndrome coronavirus 2, SARS-CoV-2, SARS, COVID-19, 2019 novel coronavirus disease, coronavirus disease, zoonoses, viruses, coronavirus, weddings, mass gatherings, Jordan

## Abstract

In March 2020, a wedding in Jordan led to a large outbreak of coronavirus disease (COVID-19). We collected data on 350 wedding attendees, 76 who of whom developed COVID-19. Our study shows high communicability of COVID-19 and the enormous risk for severe acute respiratory syndrome 2 virus transmission during mass gatherings.

As of March 15, 2020, Jordan had only 1 confirmed case of coronavirus disease (COVID-19) (*1*). However, a wedding ceremony on March 13 led to a large outbreak of COVID-19 in northern Jordan. We believe the index case-patient was the bride’s father, who arrived in Jordan from Spain, where local disease transmission was occurring (*2*), 4 days before the wedding. We describe characteristics of confirmed COVID-19 cases, symptoms, time from exposure to symptom onset, and other clinical features.

## Methods

On March 13, a 2-hour wedding ceremony and party were held in an indoor venue designed to accommodate <400 guests. The exact number of attendees is unknown but estimated to be ≈360 based on discussions with the local health department. We identified 350 attendees and collected nasopharyngeal swabs for reverse transcription PCR (RT-PCR), regardless of the presence or absence of symptoms. Our study included persons who attended the wedding or had close contact with attendees and tested positive for severe acute respiratory syndrome coronavirus 2 (SARS-CoV-2), the causative agent of COVID-19. We collected data by using electronic medical records and direct phone calls with confirmed cases or their legal guardians. We performed RT-PCR on samples from 350 possible cases within 4 weeks of exposure at the wedding.

We collected demographic data, including age and gender; each person’s general health status; symptoms at time of diagnosis; time from exposure to symptom onset or diagnosis, if asymptomatic; severity of symptoms, if present; and outcomes for confirmed cases. We analyzed data by using SPSS Statistics 21 (IBM, https://www.ibm.com) and used descriptive statistics to calculate means, medians, and ranges or counts and percentages. Jordan University of Science and Technology, Irbid, provided IRB approval (no. 233-2020)

The presumed index case-patient, the bride’s father, is a 58-year-old man. He developed fever, cough, and a runny nose 2 days before the wedding. On March 15, he went to an emergency department and tested positive for SARS-CoV-2. He had no known exposure to persons with COVID-19 in Spain. When he arrived in Jordan, he had contact with his immediate family, other relatives, and the groom during the 4 days before the wedding.

Four weeks after the wedding, in addition to the index case-patient, 85 persons with a history of exposure related to the wedding tested positive for SARS-CoV-2. Of these, 76 (89.4%) attended the wedding; 9 (10.6%) did not attend the wedding but were close contacts of confirmed cases from the wedding. All confirmed COVID-19 case-patients were admitted to the hospital and strictly monitored daily, following national policy for the care of persons with confirmed cases. 

Among 76 wedding attendees who tested positive for SARS-CoV-2, 40 (52.6%) were symptomatic and 36 (47.4%) were asymptomatic at diagnosis (Table). All were detected during surveillance performed by the public health authority of the ministry of health. One case-patient was pregnant and delivered a healthy full-term baby on the second day of hospital admission. Samples collected from the baby at birth and 48 hours of age tested negative for SARS-CoV-2 by RT-PCR. 

**Table:** Demographic and clinical features of 76 persons with confirmed coronavirus disease who attended a wedding, Jordan

Characteristics	No. (%)
Sex	
M	30 (39.5)
F	46 (60.5)
Relatives and friends of groom*	44 (57.9)
Relatives and friends of bride	32 (42.1)
Demographics	
Children <18 y	17 (22.4)
Adults >18 y	59 (77.6)
Age group, median 27 (range 2–80) y	
0–9	3 (3.9)
10–19	17 (22.4)
20–29	21 (27.6)
30–39	6 (7.9)
40–49	12 (15.8)
50–59	11 (14.5)
>60	6 (7.9)
Symptoms at time of diagnosis	
Yes	40 (52.6)
No	36 (47.4)
Concurrent conditions†	
Yes	15 (19.8)
No	61 (80.2)

*Including the groom. †Concurrent conditions included hypertension, diabetes mellitus, hypothyroidism, ischemic heart disease, and cancer.

Among symptomatic case-patients, the most common signs and symptoms were cough (70%), fever (60%), runny or congested nose (52.5%), headache (35%), sore throat (25%), fatigue or myalgia (17.5%), and shortness of breath (12.5%). Most (38) had mild symptoms, but 2 case-patients had serious or critical conditions. One, an 80-year-old woman with breast cancer, developed progressive pneumonia and respiratory failure and died 2 weeks after admission to the hospital for monitoring. We noted no statistical difference in the presence of concurrent conditions between symptomatic (15%) and asymptomatic (19%) case-patients (p = 0.76). The median time from exposure at the wedding and symptom onset was 5 days (range 2–13 days).

All 9 confirmed case-patients who did not attend the wedding were household contacts of wedding attendees. Among those, only 4 were symptomatic and developed initial symptoms 9, 11, 16, and 19 days after the wedding. The other 5 cases were asymptomatic and were detected during government surveillance. No further cases were confirmed at 4 weeks after the wedding.

## Conclusions

COVID-19 is a serious pandemic disease and a public health threat. Our study shows a relatively high communicability of SARS-CoV-2. Among 350 identified wedding attendees, 76 tested positive by RT-PCR, an attack rate of 22%. Furthermore, those who were infected during the primary encounter became new sources for disease transmission, which was evident by confirmed cases among household contacts. The estimated basic reproduction number (R_0_) for COVID-19 is 2–3.5, which means that 1 infected person can transmit the disease to 2–3 other susceptible persons (*3*,*4*). However, in closed and crowded social gatherings, the transmission rate can be much higher, as evidenced by this investigation. In Jordan, close physical contact, such as same-sex hugging, cheek-kissing, and hand shaking, are traditional wedding practices that convey congratulations to the host families. Also, immediate family members, especially parents of the bride and the groom, usually stand at the entrance of the wedding hall to receive congratulations from all guests. These factors, in addition to crowded dancing and close face-to-face communication, likely contributed to the large number of infections from this wedding.

We also noted a high rate (47.4%) of asymptomatic carriers among those infected. Asymptomatic carriers have been described in the literature (*5*–*7*), but their exact proportion in the community remains unknown. In addition, evidence suggests that asymptomatic SARS-CoV-2 carriers can transmit the virus to their contacts during the incubation period, which makes containing the disease more difficult (*8*,*9*). In our study, symptomatic case-patients reported symptom onset 2–13 days (median 5 days) after exposure, which coincides with the reported incubation period for COVID-19 (*3*). However, new data suggests a longer incubation period of <24 days is possible (*4*).

Our study has some limitations. We were not able to determine the total number of wedding attendees, which might have led us to overestimate the infectivity rate from this social gathering. Surveillance of asymptomatic persons might have missed some cases if viral shedding had not yet started or had ended before RT-PCR testing, which potentially caused us to miss some cases.

Before the outbreak from this wedding sparked a surge in COVID-19 disease, only 1 case, which was imported, had been reported in Jordan in early March. By April 10, the number of confirmed cases from the wedding constituted 24% of all COVID-19 cases in Jordan (*10*). The country has been under lockdown since March 14, and the city of Irbid, where the outbreak occurred, was isolated from the rest of the country to control the disease. No new wedding-related cases were detected by the fourth week after the event. Apart from the 1 fatality, all patients either recovered or greatly improved, and most were discharged from the hospital. 

Our study highlights the enormous risk for SARS-CoV-2 infection during mass social gatherings and the role such gatherings can play in the spread of COVID-19. In addition, it provides further evidence of asymptomatic SARS-CoV-2 transmission among secondary contacts. Communities should continue to discourage large gatherings, identify and test social contacts, and isolate confirmed cases to help control the global COVID-19 pandemic. 
